# Delayed Diagnosis of Disseminated Invasive Aspergillosis with Purulent Myocarditis in an Immunocompromised Host

**DOI:** 10.3390/idr16060093

**Published:** 2024-11-30

**Authors:** Mark Londema, Maarten W. N. Nijsten, Joost Bart, Janke S. Wiegersma, Bhanu N. M. Sinha, Douwe F. Postma

**Affiliations:** 1 Department of Critical Care, University Medical Center Groningen, University of Groningen, 9713 GZ Groningen, The Netherlandsm.w.n.nijsten@umcg.nl (M.W.N.N.); 2 Department of Pathology, University Medical Center Groningen, University of Groningen, 9713 GZ Groningen, The Netherlands; j.bart@umcg.nl; 3 Department of Internal Medicine and Nephrology, University Medical Center Groningen, University of Groningen, 9713 GZ Groningen, The Netherlands; j.s.wiegersma@umcg.nl; 4 Department of Medical Microbiology and Infection Prevention, University Medical Center Groningen, University of Groningen, 9713 GZ Groningen, The Netherlands; b.sinha@umcg.nl; 5 Department of Internal Medicine and Infectious Diseases, University Medical Center Groningen, University of Groningen, 9713 GZ Groningen, The Netherlands

**Keywords:** invasive aspergillosis, myocarditis, immunocompromised host

## Abstract

**Introduction:** Invasive aspergillosis (IA) is an opportunistic fungal infection that typically occurs in the immunocompromised host and is associated with severe morbidity and mortality. Myocardial abscess formation is seldomly described. **Detailed Case Description:** We present a case of IA with purulent myocarditis. The patient was on long-term high-dose corticosteroid and mycophenolate mofetil therapy for severe lupus nephritis. After multiple visits to his general practitioner and nephrologist for general malaise, he was admitted to our hospital with visual complaints. Within several days, he developed atrial fibrillation, respiratory insufficiency, and, finally, a decreased level of consciousness. After admission to the intensive care unit, the broncho alveolar lavage (BAL) fluid galactomannan (GM) index was normal, but the serum GM index was severely elevated. Despite initiation of antifungal therapy, the patient passed away shortly thereafter. Autopsy revealed massive intracranial hemorrhage and disseminated IA affecting the lungs, brain, and myocardium, with macroscopic myocardial abscess formation. **Discussion:** This classic case of diagnostic uncertainty illustrates how invasive fungal infections can progress to disseminated disease while showing nonspecific symptoms only. It emphasizes the importance of vigilance for opportunistic fungal infections in a growing category of immunocompromised patients. **Conclusion:** Clinicians should have a low threshold of suspicion for fungal infections in patients on combination immunosuppressive medication, such as high-dose corticosteroid therapy in combination with T-cell inhibitors like MMF.

## 1. Introduction

Invasive aspergillosis (IA) is an opportunistic fungal infection caused by species of the filamentous fungus *Aspergillus*. It typically occurs in immunocompromised hosts and is associated with severe morbidity and mortality [1]. Inoculation occurs by airborne spores, which is why the lungs are most commonly affected. Subsequently, spores can germinate into hyphae, leading to tissue invasion, infection, and vascular involvement. Aspergillosis can further progress to disseminated disease, through hematogenous spread, and potentially involve the central nervous system, heart, liver, kidneys, thyroid, and virtually any other tissue [2,3].

Diagnosis can be challenging since symptoms are nonspecific and distinctive radiological features are frequently absent. IA is usually diagnosed through imaging, histopathology, antigen testing, PCR, or culture of specimens from the infected site in immunocompromised hosts [2]. The primary treatment consists of antifungal treatment with azoles (e.g., voriconazole), although resistance to azoles can occur. Surgery may be useful in certain severe focal pulmonary of sinusoidal infections [3].

Here, we present a case of disseminated *Aspergillus fumigatus* infection with pulmonary, cerebral, and myocardial abscess formation. The extensive myocardial inflammation and abscess formation was only identified during post-mortem examination. The patient was on long-term high-dose prednisolone and mycophenolate mofetil (MMF) therapy for severe lupus nephritis. We provide a concise review of *Aspergillus* myocarditis cases reported in the literature, where macroscopic abscess formation was rare, summarize the current knowledge on pathophysiology, and discuss diagnostic strategies in the discussion.

## 2. Detailed Case Description

A 73-year-old male was admitted to our hospital with visual disturbances expressed as bilateral blurred vision and black spots. He had been using prednisolone 60 mg once daily and mycophenolate mofetil 1 g twice daily for five months for systemic lupus erythematosus (SLE) with severe nephritis. Additionally, he received furosemide 40 mg once daily for fluid retention related to severe renal insufficiency (estimated glomerular filtration rate 12 mL/min) and amlodipine 5 mg once daily for hypertension.

His visual complaints developed over two days. He visited his general practitioner and nephrologist several times in the past months for general malaise, which was attributed to the SLE. General physical examination was unremarkable except for a trace of pitting edema of the lower extremities. Vital signs were within normal range. Ocular examination showed bilateral panuveitis with chorioretinal infiltrates. The C-reactive protein (CRP) was 100 mg/L, and leukocyte count was 15.4 × 10^−9^/L. Prior CMV serology was unknown. After diagnostic anterior chamber puncture, he was started on ganciclovir 5 mg/kg twice daily, prednisolone eye drops, and atropine eye drops for a tentative diagnosis of cytomegalovirus (CMV) retinitis. 

The next day, he developed dyspnea and worsening peripheral edema. An electrocardiogram (ECG) revealed new onset atrial fibrillation (AF) with a ventricular rate of 100/min without signs of ischemia. A chest computerized tomography (CT) scan showed signs of interstitial pulmonary edema with interlobular septal thickening, bilateral pleural effusions, diffuse ground glass abnormalities, and a trace pericardial effusion. There were some smaller bilateral patchy and nodular consolidations. CMV serology was negative. PCR tests for CMV, varicella zoster virus, and herpes simplex virus on the aqueous humor were also negative. Heart failure due to a combination of administered intravenous fluids, new onset atrial fibrillation, and community-acquired pneumonia was suspected. His furosemide dose was increased to 80 mg once daily, and he was started on ceftriaxone 2G once daily and a therapeutic dose of low molecular weight heparin, adjusted for renal function (nadroparin 7600 IE s.c. once daily). Bronchoalveolar lavage (BAL) was planned for the next day to investigate the possibility of opportunistic infections such as invasive fungal disease.

However, on day three after admission, the patient was transferred to the intensive care unit for respiratory insufficiency and a decreased level of consciousness. Routine laboratory investigation showed leukocytes 14.0 × 10^−9^/L, CRP 332 mg/L, creatine kinase 204 U/L (reference interval (RI) < 200 U/L), and cardiac troponin-T 3258 ng/L (cTnT; RI < 14 ng/L). The ECG revealed persistent AF without signs of ischemia or other pathologic findings. The patient was intubated immediately followed by BAL. The BAL galactomannan (GM) index was 0.8 (RI < 1.0), whereas the serum GM index turned out to be 5.9 (RI < 1.0). The patient was started on voriconazole 6 mg/kg i.v. twice daily and liposomal amphotericin B 3 mg/kg once daily for suspected IA.

After cessation of sedation, he remained comatose. A CT scan of the brain showed massive intraparenchymal hemorrhage with signs of herniation. In the absence of further therapeutic options, treatment was discontinued, and the patient passed away soon thereafter. 

**Figure 1:**
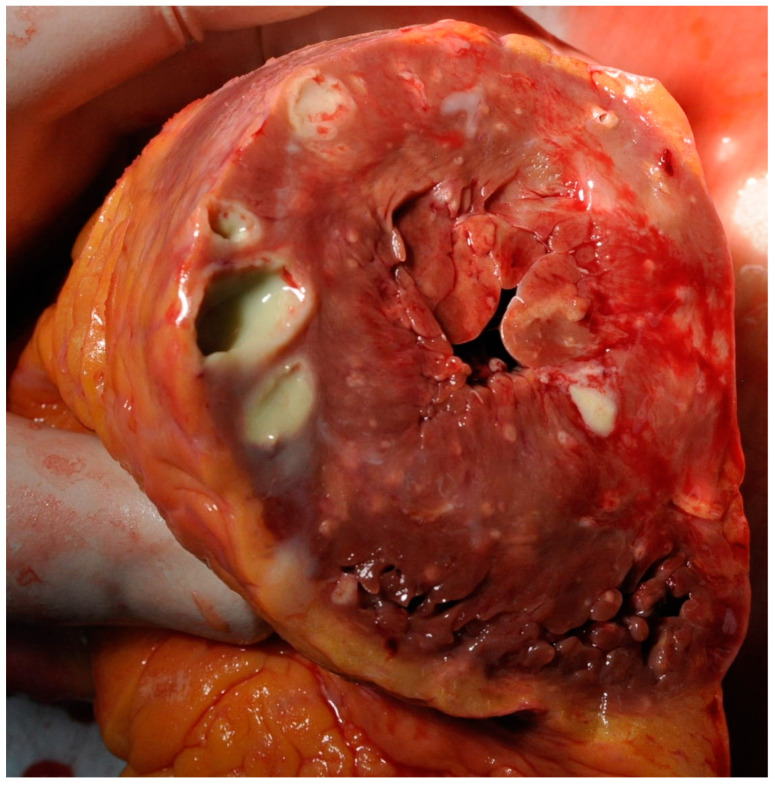
Transverse section through both ventricles, showing multiple myocardial Aspergillus abscesses.

**Figure 2:**
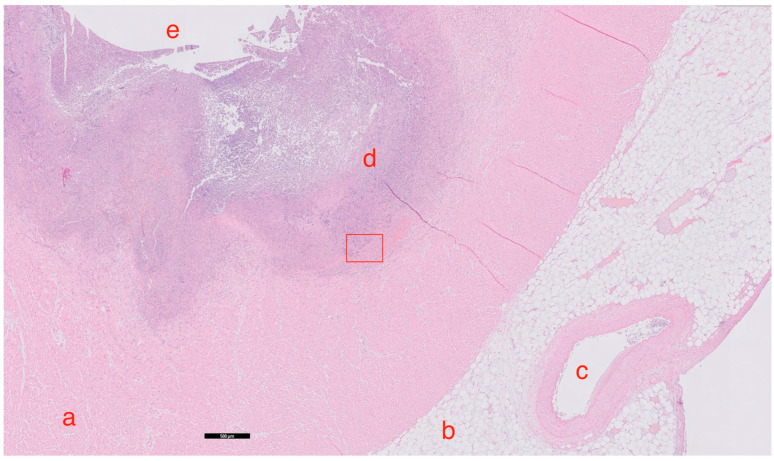
Hematoxylin and eosin staining. Black bar represents 500 μm. Micrograph of the heart, demonstrating heavy intramyocardial inflammation with abscess formation, caused by Aspergillus fumigatus. a: myocardium; b: epicardial fatty tissue; c: lumen of a branch of the coronary arterial system; d: inflammatory infiltrate and abscess wall; e: abscess cavity; rectangle: depicts area that is presented in Figure 3.

**Figure 3:**
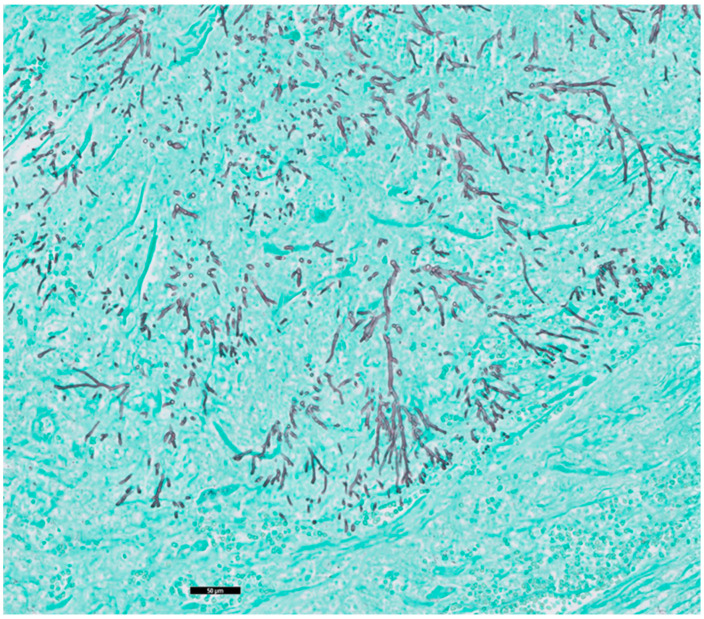
Grocott silver staining. Black bar represents 50 μm. Detailed image of the abscess wall, demonstrating the branching Aspergillus hyphae.

**Figure 4:**
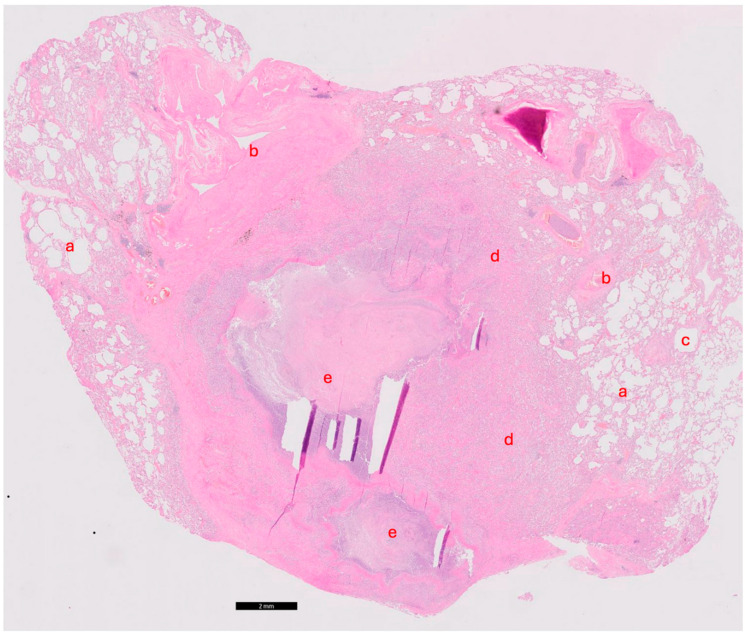
Hematoxylin and eosin staining. Black bar represents 2 mm. Micrograph of the lung demonstrating abscess with Aspergillus fumigatus. a: (alveolar) lung parenchyma; b: artery; c: vein; d: inflammatory infiltrate; e: abscess also depicted in Grocott stain in Figure 5.

**Figure 5:**
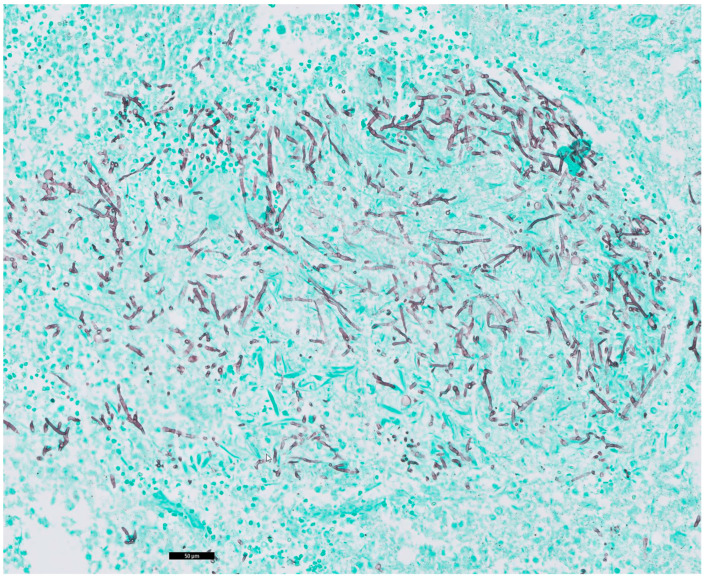
Grocott silver staining. Black bar represents 50 μm. Detailed image of the lung abscess with abundant branching Aspergillus hyphae.

Autopsy was performed as requested by the treating physicians with permission from the relatives. Gross cardiac examination revealed multiple intramyocardial abscesses (Figure 1). Microscopically, multiple inflammatory foci in a vasculocentric pattern with abscess formation were found (Figure 2). The presence of multitudes of branching hyphae was compatible with Aspergillus fumigatus (Figure 3). The coronary arteries appeared normal. There were no signs of ischemia and no valvular vegetations. In the lungs, abscesses were found in both lower lobes (Figure 4 and Figure 5). Histological examination revealed a bronchocentric pattern of fungal infiltration, suggestive for aerogenic entrance of the fungus. Examination of the brain showed intraventricular and pericerebellar hemorrhages and signs of suprafalcine and tentorial herniation. In the cerebrum, cerebellum, and brainstem, perivascular micro-abscesses were found, and Aspergillus hyphae could be demonstrated on microscopic examination. Abscess cultures from the heart, lungs, and brain were all positive for Aspergillus fumigatus.

## 3. Discussion

The increasing use of immunosuppressive medication has contributed to a rise in opportunistic fungal infections, like IA, over the last decades [4]. Data on the specific risk of IA in patients treated for SLE are limited, but the estimated incidence ranges from 0.5 to 2.1% [5]. Likely, the risk for opportunistic infection is related to the disease severity of SLE and, subsequently, intensity of (immunosuppressive) treatment. This is demonstrated in a recent observational cohort study from Taiwan and the USA showing an increased rate of severe infections in patients with lupus nephritis compared to extra-renal lupus only [6]. Similar observations in the literature have led to the conclusion that severe infections, next to disease activity and cardiovascular disease, are one of the main causes of SLE mortality [7,8]. 

Myocarditis is a seldomly reported complication of IA. A literature search revealed 12 well-described cases of IA with myocarditis proven by culture or histopathological examination (Table 1) [9,10,11,12,13,14,15,16,17,18,19]. In all cases, the outcome was fatal, and an autopsy was performed. None of the reports describe BAL or serum GM index values and/or c-TnT levels. Other organ systems were almost always involved, a sign of hematogenous dissemination. Affected organs included lungs, liver, brain, kidneys, spleen, thyroid gland, adrenal gland, and skin. Pulmonary involvement could not be demonstrated on autopsy in 3 out of 12 cases [12,14,16]. All cases had a previous history of immunocompromise.

Antifungal immunity is still incompletely understood [20]. Innate immune cells, such as neutrophils and macrophages, are primary contributors to resistance against fungal infections through phagocytosis. Macrophages also contribute to activation of the adaptive cellular immune response by their function as antigen presenting cells (APCs) [21]. In the adaptive immune response, T-helper 1 (TH1) cells play an important role in the prevention of IA by production of pro-inflammatory cytokines such as interferon gamma and TNF-alpha [22,23,24]. Corticosteroids have suppressive effects on almost all lymphoid cells and the process of phagocytosis [25]. Treatment with high-dose corticosteroids (i.e., ≥0.3 mg/kg for ≥3 weeks over the past 60 days) is generally considered to be an independent risk factor for IA [2]. MMF is a suppressor of B and T lymphocyte proliferation, including TH1 cells, by inhibition of nucleotide synthesis by inhibition of type 2 inosine monophosphate dehydrogenase [26]. The suppression of both the innate and the adaptive immune system resulting from taking both corticosteroids and MMF, leads to an increased risk of developing IA. Most patients with these risk factors do not develop IA. As such, interactions between host genetics, immune responses, and pathogens probably contribute to the risk of developing IA [20]. These contributing factors are probably also involved in the scope of dissemination of IA. Macroscopic abscess formation is a seldomly reported complication of IA. This could also be due to diagnostic delay since the patient had symptoms of general malaise for a prolonged period of several months in which he visited a neurologist and his primary care physician. Similar cases of disseminated infections with different opportunistic pathogens in SLE patients are reported [27,28].

Early IA diagnosis probably leads to better treatment outcomes, which is why GM antigen detection is recommended as an important diagnostic tool. GM is a component of the cell wall of several molds including *Aspergillus* species that can be detected in BAL fluid and serum. In the patient we describe, BAL and serum GM indices were 0.8 and 5.9, respectively. For both BAL and serum, >1.0 is recommended as a cut-off value [2]. Although the BAL fluid GM index has been shown to have greater sensitivity for detecting IA as compared to serum GM index [29], our report illustrates the usefulness of the serum GM index determination in detecting disseminated disease. In this case, the substantially elevated serum GM index was highly suggestive of invasive fungal disease. After our patient had deceased, the BAL culture and *Aspergillus* PCR also came back positive. This illustrates that the combined use of tests for different Aspergillus targets (GM antigen testing and fungal DNA) can potentially improve the diagnosis, if PCR is readily available [30]. Other diagnostic tests such as a serum beta-D-glucan assay, 18S rRNA PCR, or plasma, and whole-blood based PCR panels are not available on a daily basis in most hospitals [30].

In addition, we found that c-TnT levels can be elevated in *Aspergillus* myocarditis. C-TntT is a biomarker that can be elevated in a variety of ischemic and non-ischemic cardiac disorders including infectious myocarditis [31,32]. An elevated c-TnT in the setting of (a suspicion of) IA may prompt investigations for cardiac involvement.

## 4. Conclusions

In conclusion, we describe a patient with disseminated IA with myocarditis and multiple myocardial abscesses with a fatal outcome. This classic case of diagnostic uncertainty emphasizes the importance of vigilance for opportunistic fungal infections in a growing category of immunocompromised patients. It illustrates how these infections can progress to advanced disseminated disease while a patient is showing mild and nonspecific symptoms only. Clinicians should have a low threshold of suspicion for fungal infections in patients on combination immunosuppressive medication, such as high-dose corticosteroid therapy in combination with T-cell inhibitors like MMF.

## Figures and Tables

**Table 1:** Reported cases of proven *Aspergillus* myocarditis.

Ref.	History	Immunosuppressive Medication	Antifungal Treatment	Cardiac Imaging	Myocarditis Diagnosis Confirmed by	Other Organ Systems Affected on Autopsy
[9]	Eosinophilic granulomatosis with polyangiitis	Long-term prednisone and rituximab	None	TTE: reduced left ventricular function, no abscesses seen	Abscess culture	Lungs
[10]	Recent liver transplantation	Mycophenolate mofetil Tacrolimus Corticosteroids	Caspofungin Amphotericin B	CT and MRI: suspected intramyocardial abscess	Abscess culture	Lungs, liver, kidney, skin
[11]	Acute myeloid leukemia	Recent course of cytarabine chemotherapy	Amphotericin B Micafungin	TTE: decreased wall motion (not specified)	Fungal hyphae on microscopy	Lungs
[11]	Rheumatoid arthritis Chronic renal failure Pancytopenia	Long-term methotrexate	None	None	Fungal hyphae on microscopy	Lungs, spleen, kidneys
[12]	HIV (CD4 79/mm^3^)	None	Caspofungin Voriconazole	TTE: mitral valve leaflet vegetation	Fungal hyphae on microscopy	Kidneys, adrenal gland
[13]	Bronchiectasis	Long-term inhalation corticosteroids and 1 week of prednisone	None	None	Fungal hyphae on microscopy	Lungs
[14]	Chronic obstructive pulmonary disease Right-sided heart failure	Methylprednisolone 120 mg OD for 3 weeks	None	TTE: non dilated hypokinetic left ventricle	Fungal hyphae on microscopy	None
[15]	Treatment resistant severe ulcerative colitis	High-dose prednisone and 6-mercaptopurine, duration not specified	None	TTE: pericardial effusion and regional hypokinesia of the left ventricle	Fungal hyphae on microscopy	Lungs, kidneys, liver, thyroid, spleen, brain
[16]	HIV (CD4 12/mm^3^)	Corticosteroids for suspected PJP, duration not specified	None	TTE: mobile, globular masses in the left ventricular cavity	Fungal hyphae on microscopy	Kidney, brain
[17]	Persisting arrhythmias following recent coronary artery bypass surgery	3 weeks of methylprednisolone	None	None	Fungal hyphae on microscopy	Lungs
[18]	Recent surgery for dissecting aortic aneurysm	1 week of hydrocortisone	Amphotericin B	None	Fungal hyphae on microscopy	Lungs, kidneys, brain
[19]	Lymphosarcoma	8 days of high dose prednisone	None	None	Fungal hyphae on microscopy	Lungs, kidneys, thyroid, brain

Abbreviations; TTE: transthoracic echocardiogram; CT: computerized tomography; MRI: magnetic resonance imaging.

## Data Availability

The original contributions presented in the study are included in the article, further inquiries can be directed to the corresponding author.
